# Effects of steam sterilization on reduction of fungal colony forming units, cannabinoids and terpene levels in medical cannabis inflorescences

**DOI:** 10.1038/s41598-021-93264-y

**Published:** 2021-07-07

**Authors:** Shachar Jerushalmi, Marcel Maymon, Aviv Dombrovsky, Rafi Regev, Ze’ev Schmilovitch, Dvora Namdar, Nurit Shalev, Hinanit Koltai, Stanley Freeman

**Affiliations:** 1 Institute of Plant Protection, Agriculture Research Organization (ARO), The Volcani Center, 7505101 Rishon Lezion, Israel; 2 The Robert H. Smith Faculty of Agriculture, Food and Environment, The Hebrew University of Jerusalem, 7610001 Rehovot, Israel; 3 Institute of Agricultural Engineering, Agriculture Research Organization (ARO), The Volcani Center, 7505101 Rishon Lezion, Israel; 4 Institute of Plant Sciences, Agriculture Research Organization (ARO), The Volcani Center, 7505101 Rishon Lezion, Israel

**Keywords:** Microbiology, Plant sciences

## Abstract

Medical cannabis (MC) production is a rapidly expanding industry. Over the past ten years, many additional phytocannabinoids have been discovered and used for different purposes. MC was reported beneficial for the treatment of a variety of clinical conditions such as analgesia, multiple sclerosis, spinal cord injuries, Tourette's syndrome, epilepsy, glaucoma, Parkinson disease and more. Yet, there is still a major lack of research and knowledge related to MC plant diseases, both at the pre- and postharvest stages. Many of the fungi that infect MC, such as *Aspergillus* and *Penicillium* spp., are capable of producing mycotoxins that are carcinogenic, or otherwise harmful when consumed, and especially by those patients who suffer from a weakened immune system, causing invasive contamination in humans. Therefore, there are strict limits regarding the permitted levels of fungal colony forming units (CFU) in commercial MC inflorescences. Furthermore, the strict regulation on pesticide appliance application in MC cultivation exacerbates the problem. In order to meet the permitted CFU limit levels, there is a need for pesticide-free postharvest treatments relying on natural non-chemical methods. Thus, a decontamination approach is required that will not damage or significantly alter the chemical composition of the plant product. In this research, a new method for sterilization of MC inflorescences for reduction of fungal contaminantstes was assessed, without affecting the composition of plant secondary metabolites. Inflorescences were exposed to short pulses of steam (10, 15 and 20 s exposure) and CFU levels and plant chemical compositions, pre- and post-treatment, were evaluated. Steam treatments were very effective in reducing fungal colonization to below detection limits. The effect of these treatments on terpene profiles was minor, resulting mainly in the detection of certain terpenes that were not present in the untreated control. Steaming decreased cannabinoid concentrations as the treatment prolonged, although insignificantly. These results indicate that the steam sterilization method at the tested exposure periods was very effective in reducing CFU levels while preserving the initial molecular biochemical composition of the treated inflorescences.

## Introduction

The Medical Cannabis (MC) industry is rapidly expanding; in 2018 more than 2 million people were estimated consuming MC for various medical purposes in the United States alone^1^. In Canada, there has been a 24% increase in patients reported to be using MC during the years 2015 to 2017. MC research has advanced at a faster rate than that of regular medical research, indicating that demand for the product has risen in parallel to that of patient's requiring MC for medical purposes^2^.

One particular issue that has not been thoroughly investigated is the subject of hazards of microbial exposure due to MC consumption. This is evident since less than 1% of cannabis-related research found in the web of science was related to pathogens, mycotoxins, spoilage, biocontrol or phytopathology disciplines^2^. Research that was conducted on the subject indicated that potential health hazards existed when patients were exposed to different types of fungal spores present in MC inflorescences^3–5^. Various fungi such as *Alternaria* spp., *Aspergillus* spp., *Fusarium* spp., and *Penicillium* spp. are known to produce mycotoxins^6–8^. Mycotoxins are stable small molecules produced by certain fungi, some of which are highly toxic and even carcinogenic to humans and other mammals. The presence of these fungi in MC may expose patients consuming cannabis for medical purposes to mycotoxins and their adverse effects^8^, such as invasive pulmonary Aspergillosis caused by exposure to *Aspergillus* spp.^9–11^.

Thus, there is a basic requirement for safe decontaminated MC inflorescences provided to patients. In Israel, the permitted total yeast and mold (TYM) colony forming unit (CFU) levels for MC inflorescence set by the Israeli Medical Cannabis Agency (IMCA) is less than 2000 TYM CFUs/g of dried inflorescence^12^. Notably, the natural microbial flora levels colonizing commercial inflorescences in cultivation facilities is commonly much higher than the permitted total levels, requiring a post-harvest treatment^12^. Effective nonchemical post-harvest sterilization methods including ionizing and cold plasma radiation have been reported^12^. While these methods were proven effective at CFU reduction they are costly to apply^12^.

Moreover, the most active compounds in cannabis, i.e., cannabinoids and terpenes, tend to degrade under heat and U.V. radiation exposure, making certain traditional sterilization methods such as autoclaving irrelevant due to their negative side-effects^13–15^. The two most common and well-known cannabinoids are tetrahydrocannabinol (THC) and cannabidiol (CBD), but there are well over 150 additional phytocannabinoids found in cannabis strains^14,16–18^. Nowadays, there are a multitude of studies indicating the health benefits and the synergistic effects of these other cannabinoids, proving them to be as important as THC and CBD^17,19^. One of these cannabinoids that has been neglected is cannabigerol (CBG), shown in various *in-situ* tests to assist in different bowel conditions such as inflammatory bowel disease (Borrelli et al., 2013; 2014).

With respect to terpenes and the relation to health, it is still unclear to what degree these compounds affect the biological activity of MC. Several studies claim that according to the "entourage effect", the combination of specific terpenes with certain cannabinoids plays a crucial role in the medical effect of MC^14,22–24^. Either way, hundreds of different terpenes have been identified in different MC cultivars and it is paramount not to substantially affect their composition for MC fragrance and medical purposes^14,23^. In this study, all terpenes, terpene alcohols, terpene aldehydes and other terpenoidic forms will be referred to as “terpenes” when generally discussed.

Previously we reported on the use of beta radiation (E-beam) and cold plasma for MC inflorescence sterilization^12^. In this research we tested an additional method utilizing steam sterilization for decontamination of total yeasts and molds (TYM). Heating the surface of a fresh or processed agricultural product can be used to eliminate various pests and pathogens, provided the treatment does not damage the crop or its bioactive compounds^25^. Complete disinfection was achieved by a short and uniform heating over the entire surface of carrot^25^ using equipment that was developed at the Institute of Agricultural Engineering of ARO (Israeli patent No. 196828).

In this study, MC inflorescence sterilization with short exposures of steaming was evaluated using prototype equipment developed in the ARO, Volcani Center. The steaming treatments were analyzed for microbial contamination and full chemical profiles of cannabinoid and terpene compositions of the treated inflorescences, to ensure the quality of the consumed product.

## Results and discussion

### Effect of steam sterilization on fungal decontamination of dried noncommercial and commercial MC inflorescences

Steam sterilization of noncommercial MC inflorescences in both experiments resulted in a significant reduction of TYM CFU levels to negligible values (Table 1). In the first experiment, CFU levels were reduced from 10^4.4^ ± 1.02 in the untreated control to 0, 2 and 0 CFUs/g inflorescence, for the 10, 15 and 20 s steam exposure treatments, respectively (Table 1). In the second experiment, TYM CFUs levels were reduced from 10^3.05^ ± 1.15 CFUs/g inflorescence to 0 in all three treatments (Table 1). In both experiments, the major fungal species present in the control treatment plates were *Aspergillus* and *Penicillium* spp.

**Table 1:** Colony forming units **(**CFU) levels in MC inflorescences.

Treatment^a^	Noncommercial inflorescences		Commercial inflorescences		
	Exp. 1	Exp. 2	Exp. 1	Exp. 2	Exp. 3
Control	4.40 ± 0.01^b^	3.05 ± 0.06	4.16 ± 0.08	5.58 ± 0.02	3.23 ± 0.05
10 s	0 ± 0	0 ± 0	0 ± 0	0 ± 0	0 ± 0
15 s	0.31 ± 0.31	0 ± 0	0 ± 0	0 ± 0	0 ± 0
20 s	0 ± 0	0 ± 0	0 ± 0	0 ± 0	0 ± 0

^a^CFU survival values for noncommercial and commercial inflorescences exposed to 10, 15 and 20 s steam treatments in the different experiments.

^b^Values are presented as log 10 (CFU/g inflorescence) ± standard error.

Similarly, steam sterilization of commercial MC inflorescences was extremely effective in eliminating TYM CFUs levels to 0 CFUs/g inflorescence in all three treatments (10, 15 and 20 s) for all three experiments (Table 1). In the first and third commercial experiments, the major fungal species present in the control treatment plates was *Cladosporium* spp., while in the second commercial experiment *Alternaria* spp. and *Fusarium* spp. composed the majority of colonies in the control treatment plates.

The impact of microbial contamination on quality of MC products cannot be overly stated, as many studies and experts claim, whereby, the definition of a "quality product" in this respect refers to microbial decontamination among other issues^1,2,26^.

With regards to “disease-free” MC products, the results of steam sterilization for both commercial and noncommercial inflorescences proved to be very effective in TYM CFU reduction in MC inflorescences, to levels below detection, even at the shortest exposure time of 10 s (Table 1). These results were as effective as various other sterilization methods that we previously evaluated, i.e. e-beam and cold plasma sterilization^12^.

### Effect of steam sterilization on terpene content in noncommercial inflorescences

In the first experiment applied to noncommercial inflorescences, monoterpene profiles changed slightly with prolonged exposure intervals (Table 2). For example, D-limonene concentrations measured 4.19%, 4.08% and 2.95% in the untreated control, 15 and 20 s steaming exposures treatments, respectively (Table 2). Similarly, linalool concentrations measured 4.73%, 4.28% and 2.88% in the untreated control, 15 and 20 s steaming exposure treatments, respectively (Table 2).

**Table 2:** List of terpenes and terpenoids and their relative amounts detected by GC–MS from the first noncommercial experiment.

Compound name	Terpene / total extract (%)		
	Control	15 s steaming treatment	20 s steaming treatment
α-Pinene	0.19	0.50	–^a^
β-Pinene	0.66	–	0.36
β-Myrcene	3.39	2.54	1.46
D-Limonene	4.19	4.08	2.95
Linalool	4.73	4.28	2.88
Fenchol	3.23	3.65	3.03
trans-2-Pinanol	2.01	2.49	2.06
β-Ocimene	–	–	2.43
3-Carene	–	–	0.43
endo-Borneol	–	0.95	0.68
α-Terpineol	2.58	2.94	2.41
cis-α-Bergamotene	0.26	–	0.25
Caryophyllene	10.56	13.54	14.97
γ-Elemene	0.56	0.57	0.60
trans-α-Bergamotene	1.68	2.28	2.41
(E)-β-Famesene	2.53	3.03	2.68
α-Humulene	4.62	6.17	7.01
γ-Muurolene	0.28	–	0.32
β-Selinene	1.42	1.44	2.01
α-Selinene	–	1.61	2.05
α-Farnesene	4.10	4.57	4.47
(-)-Guaia-6,9-diene	–	–	2.09
β-Bisabolene	0.56	–	2.27
Cyclosativene	1.60	1.92	2.30
β-Sesquiphellandrene	0.44	2.04	–
Valencene	7.30	8.47	9.54
Selina-3,7(11)-diene	8.00	9.46	11.37
Germacrene B	8.60	11.37	14.97
trans-Longipinocarveol	0.88	1.17	1.34
3,5,11-Eudesmatriene	1.67	1.35	–
Eudesm-7(11)-en-4-ol	0.73	0.87	0.87
α-Gurjunene	0.46	–	0.60

^a^Concentration below detection levels.

The sesquiterpene profile also changed upon steaming, however contrary to those of the monoterpenes, i.e., an increase in concentrations was associated with increased exposure times (Table 2). For example, α-humulene concentrations measured 4.62%, 6.17% and 7.01% in the untreated control, 15 and 20 s steaming exposure treatments, respectively.

In the second noncommercial experiment, the terpene profiles were more affected by steam sterilization treatments than in the first experiment. After 15 s exposure, 12 of the 33 terpenoids initially detected in the inflorescences were absent in the extract (Table 3). After 20 s exposure, 22 of the 33 terpenoids did not recur. This indicates that 67% of the total terpenes and terpenoids produced by the plant were lost during the steaming process (Table 3). However, it is important to mention that the initial concentrations of monoterpene in those samples were relatively lower than those recovered from the inflorescences sampled in the first experiment (Tables 2 and 3). Yet, changes in monoterpenes with higher initial concentrations, such as linalool and fenchol, showed a similar pattern to that observed in the first experiment.

**Table 3:** List of terpenes and terpenoids and their relative amounts detected by GC–MS from the second noncommercial experiment.

Compound name	Terpene / total extract (%)		
	Control	15 s steaming treatment	20 s steaming treatment
α-Thujene	0.59	0.65	–^a^
α-Pinene	1.66	0.54	–
Sabinene	0.87	0.89	0.66
β-Pinene	1.62	0.59	–
( +)-3-Carene	1.89	–	–
( +)-4-Carene	0.56	0.53	–
D-Limonene	1.68	0.63	0.57
β-Thujene	0.13	–	–
Eucalyptol	–	0.60	0.77
β-Ocimene	1.07	–	–
γ-Terpinene	0.82	0.69	–
Sabinene hydrate	0.73	0.61	–
2-Carene	3.95	0.68	–
Linalool	3.55	2.76	2.98
Fenchol	3.51	2.94	2.81
trans-2-Pinanol	2.49	2.24	2.10
endo-Borneol	1.37	1.30	1.23
Terpinen-4-ol	0.86	0.51	–
Butanoic acid, hexyl ester	0.48	–	–
L-α-Terpineol	5.34	4.94	3.96
coumaran	–	–	0.69
Citronellol	0.63	0.42	–
Caryophyllene	21.04	22.46	28.47
γ-Elemene	0.68	0.67	0.68
trans-α-Bergamotene	2.70	1.62	1.21
(E)-β-Famesene	1.70	0.88	–
α-Humulene	8.20	9.57	11.33
(-)-aristolochene	–	0.72	0.95
α-Guaiene	1.93	2.33	2.62
7-Epi-α-Selinene	1.64	1.75	2.22
(E)-γ-Bisabolene	2.26	1.92	2.39
β-Bisabolene	0.73	–	–
γ-Gurjunene	0.87	1.00	1.58
α-Gurjunene	1.49	1.33	1.97
Guaia-3,9-diene	6.27	5.77	7.27
Selina-3,7(11)-diene	6.50	5.81	7.85
γ-Elemene	3.18	3.97	4.24
Caryophyllene oxide	1.01	1.36	1.47
Myo-Inositol	0.47	7.15	–
Longifolene	1.07	1.95	1.65
α-Guaiene	0.63	1.25	–
3,5,11-Eudesmatriene	0.63	–	–
α-Bisabolol	0.44	0.86	0.96
Eudesm-7(11)-en-4-ol	0.58	0.65	0.86
n-Hexadecanoic acid	–	1.16	1.42
Phytol	0.50	–	1.48
Linoelaidic acid	0.93	2.20	1.48
9,12,15-Octadecatrienoic acid	0.77	2.08	2.11

^a^Concentration below detection levels.

Sesquiterpene patterns such as β-caryophyllene and α-humulene, in the second experiment, were more stable than the monoterpene profile, and similar to that in the first experiment, where an increase in concentrations was demonstrated for longer exposure times (Tables 2 and 3). For example, caryophyllene concentrations increased to 22.46% and 28.47% in the 15 and 20 s steam sterilization treatments, compared to 21.04% in the untreated control, respectively (Table 3).

Certain monoterpenes constantly appeared in the profile only after the inflorescences were exposed to steam sterilization. These post-treatment terpenes include β-ocimene, 3-carene and endo-borneol that were detected only after 20 s steaming (Tables 2 and 3). Some degradation by-product monoterpenes such as endo-borneol were detected after 15 s exposure, and their concentration decreased after 20 s treatment (Table 2). For example, the relative amount of endo-borneol increased from 0% in the untreated control to 0.95% after 15 s steaming but decreased to 0.68% after 20 s exposure (Table 2).

Two sesquiterpenes, guaia-6,9-diene and α-selinene were consistently absent in the untreated inflorescences, but were detected in the treated samples (Table 2). Their relative amounts were increased with increased steam exposure times, i.e., higher amounts of guaia-6,9-diene and selinene were detected after 20 s of steaming compared to that of the 15 s exposures and absence in the initial inflorescences.

### Effects of steam sterilization on terpene content in commercial inflorescences

In general, exposure of the commercial inflorescences to 20 s steaming caused a similar decrease in the monoterpene profiles, compared to that of the noncommercial samples (Tables 4, 5 and 6). In the second commercial experiment, exposures of 15 s steaming resulted in a slight increase in the monoterpene concentrations compared to that of the untreated control. After 20 s steaming, a decrease in monoterpene concentrations was detected compared to 15 s treatment but remained higher than that of the untreated control. For example, linalool concentrations measured 1.78%, 2.39% and 1.83% in the untreated control, 15 and 20 s treatments, respectively (Table 6). Similarly, caryophyllene concentrations measured 7.41%, 8.07% and 7.61% in the untreated control, 15 and 20 s treatments, respectively (Table 6). This indicates that a two-step degradation process occurred, first degradation of di- and sesqui-terpenes into monoterpenes, resulting in elevation in relative monoterpene quantities. Then with a further prolonged exposure, monoterpenes further degraded and/or evaporated, resulting in lower relative quantities (Tables 4, 5 and 6).

**Table 4:** List of terpenes and terpenoids and their relative amount detected by GC–MS for ethanolic extraction of inflorescences from first commercial experiment.

Compound name	%		
	Control	15 s steaming treatment	20 s steaming treatment
Heptanal	1.73	3.68	5.67
Cosmene	0.45	–	–
Carveol	0.60	1.03	–
Linalool	2.73	1.60	5.95
Nonanal	2.23	4.61	–
Fenchol	2.19	1.45	–
Camphene	1.86	1.13	–
endo-Borneol	0.76	–	–
α-Terpineol	2.22	1.95	2.52
Methenamine	1.82	3.63	6.42
Caryophyllene	7.36	6.67	7.89
trans-α-Bergamotene	1.25	–	–
cis-β-Farnesene	0.75	–	–
α-Humulene	3.19	2.60	4.31
Valerena-4,7(11)-diene	0.49	–	–
pseudoephedrine	0.41	–	–
α-Farnesene	1.11	1.92	2.97
Patchoulene	0.56	–	–
gamma-Selinene	0.61	–	–
gamma-Gurjunene	3.50	2.62	3.76
Selina-3,7(11)-diene	5.22	7.68	9.33
α-Guaiene	0.69	–	–
m-Mentha-4,8-diene	1.47	1.36	–
Guaiol	7.40	8.30	9.83
cis-Z-α-Bisabolene epoxide	1.12	–	–
δ-Selinene	13.04	14.03	16.19
β-Guaiene	2.95	2.49	–
γ-cadina-1,4-diene	1.52	–	–
Guaia-1(10),11-diene	–	1.45	–
α-Guaiene	1.96	–	–
7-epi-α-Eudesmol	14.82	17.33	–
Selina-3,7(11)-diene	0.83	–	–
Eudesmadiene	3.47	2.00	–
cis-Thujopsene	1.52	0.78	–
β-Selinene	0.56	–	–
β-Neoclovene	1.06	0.91	–
epi-Cryptomeridiol	0.55	2.00	18.89
Neophytadiene	1.19	0.93	3.34
Phytol	1.17	2.50	2.92
11,14-Eicosadienoic acid, methyl ester	0.43	0	0
Methyl 8,11,14,17-eicosatetraenoate	0.67	1.43	0
Benzanthracene methoxy	1.27	1.64	0
9-Anthracenecarboxaldehyde	1.25	2.26	0

**Table 5:** List of terpenes and terpenoids and their relative amounts detected by GC–MS from the second commercial experiment.

Compound name	Terpene / total extract (%)		
	Control	15 s steaming treatment	20 s steaming treatment
α-Pinene	0.24	0.19	0.19
Camphene	0.09	–^a^	–
β-Pinene	0.73	0.57	0.54
β-Myrcene	1.66	0.63	0.86
D-Limonene	4.25	2.89	2.97
Fenchone	0.29	0.24	0.30
Linalool	1.72	1.48	1.87
Fenchol	1.47	1.31	1.65
4-Carene	1.51	–	1.71
3-Carene	–	–	0.40
Borneol	0.43	0.44	0.54
Terpineol	1.17	1.17	1.42
(-)-Carvone	0.15	–	–
α-Ylangene	0.30	0.27	0.36
cis-Eudesma-6,11-diene	0.16	–	0.17
Caryophyllene	28.98	27.88	29.26
γ-Elemene	6.12	5.83	5.62
trans-α-Bergamotene	0.76	0.84	0.59
(-)-Guaia-6,9-diene	0.43	0.47	0.63
cis-muurola-3,5-diene	2.73	2.67	2.83
α-Humulene	8.27	8.12	8.33
β-Elemene	1.11	1.23	1.18
Germacrene D	0.20	0.20	0.26
β-Muurolene	0.20	0.29	–
β-Selinene	1.06	0.94	1.02
β-Elemene	0.34	0.33	–
7-epi-α-Selinene	1.28	1.08	1.15
Cadina-1(10),4-diene	0.82	1.01	0.93
β-Bisabolene	0.61	0.63	0.54
γ-Cadinene	2.01	1.94	2.03
δ-Cadinene	0.83	0.73	0.84
β-Guaiene	0.72	0.68	0.64
α-Guaiene	0.25	–	0.24
γ-Muurolene	0.67	0.64	0.78
β-Maaliene	0.94	0.95	0.72
Eremophila-1(10),11-diene	4.65	4.17	4.17
Selina-3,7(11)-diene	5.56	4.92	4.42
Germacrene B	13.58	13.32	12.14
γ-Gurjunene	0.13	–	0.18
Caryophyllene oxide	0.66	0.65	0.92
Guaiol	0.35	0.77	–
Cadina-1(10),4-diene	–	–	0.77
epi-γ-Eudesmol	0.65	1.11	0.54
( +)-Valencene	0.51	0.25	0.61
Longifolene	–	0.27	0.19
Ledol	0.90	1.43	1.03

^a^Concentration below detection levels.

**Table 6:** List of terpenes and terpenoids and their relative amounts detected by GC–MS from the third commercial experiment.

Compound name	Terpene / total extract (%)		
	Control	15 s steaming treatment	20 s steaming treatment
β-Pinene	0.68	0.74	0.70
D-Limonene	0.37	0.41	0.39
Linalool	1.78	2.39	1.83
Fenchol	0.80	1.11	0.82
trans-2-Pinanol	0.71	0.88	0.73
Borneol	0.54	0.64	0.55
L-α-Terpineol	1.55	1.79	1.59
8-Hydroxylinalool	0.51	–^a^	–
cis-α-Bergamotene	0.39	0.37	0.40
Caryophyllene	7.41	8.07	7.61
γ-Elemene	0.18	0.18	0.19
trans-α-Bergamotene	1.66	1.80	1.71
(E)-β-Famesene	2.62	2.75	2.69
α-Humulene	3.74	3.99	3.83
γ-Muurolene	0.245	0.26	0.25
γ-Curcumene	0.49	0.50	0.51
Eudesma-4(14),7(11)-diene	0.71	0.71	0.73
α-Gurjunene	0.44	0.44	–
α-Guaiene	0.96	1.01	1.0
α-Elemene	1.03	1.08	–
α-Selinene	0.83	0.89	0.85
(E)-γ-Bisabolene	2.95	3.25	3.03
β-Bisabolene	1.18	1.22	1.21
Sesquicineole	0.51	–	–
Guaia-6,9-diene	–	1.17	–
β-Sesquiphellandrene	0.55	0.58	–
α-Gurjunene	2.37	2.53	2.44
isoledene	7.71	–	8.94
α-Maaliene	10.50	11.11	10.78
β-Guaiene	0.55	0.49	–
Caryophyllene oxide	1.74	1.68	1.79
Guaiol	10.18	10.20	10.45
7-epi-γ-Eudesmol	12.48	13.47	12.81
α-Eudesmol	2.85	2.77	1.01
Selina-3,7(11)-diene	0.71	–	0.73
Agarospirol	0.86	0.82	0.88
Neoisolongifolene	–	2.24	2.34
Guai-1(10)-en-11-ol	9.95	10.0	10.22
α-Selinene	–	0.76	0.78
α-Bisabolol	2.66	2.59	2.73
Eudesm-7(11)-en-4-ol	1.79	1.79	1.84
Olivetol	0.67	0.62	0.68

^a^Concentration below detection levels.

In the commercial inflorescence experiments, guaia-6,9-diene and selinene (either as α- or β- isomers) were detected only after steaming, similar to that for the noncommercial samples (Table 4). In general, the 20 s steam treatment caused more damage to the monoterpenes than the 15 s exposure period. Similar to the noncommercial experiments upon exposure to steaming, the relative amounts of sesquiterpenes increased as steaming prolonged. However, it appears that the steaming exposures had little effect on monoterpene profiles in all experiments.

While steaming evidently had an effect on terpene concentrations in MC inflorescences (Tables 2, 3, 4, 5, 6), the extent varied according to exposure times and the terpenes themselves. For example, with the appearance and detection of certain monoterpenes such as β-ocimene, 3-carene and endo-borneol, post treatment may indicate that larger terpenes, such as sesquiterpenes, were degraded upon steaming. The monoterpenes that were not detected in the initial terpenoid profile of the untreated control inflorescence, are likely breakdown products of the larger terpenoids.

It appeared that certain major terpenes, considered very common in MC, such as d-limonene and linalool^27^, appeared to be moderately affected by steaming. In the first noncommercial experiment, percentages of terpenes were reduced along with the increase in steam exposure periods; while the 15 s treatment had a minor effect of 0.11% reduction in d-limonene and 0.45% reduction in linalool, the effect increased after 20 s (Table 2). However, in the second commercial experiment, the percentage of d-limonene was reduced by 1.36% and 1.28% after 15 and 20 s exposure rates respectively, compared to that of the untreated control. This inconsistency may be due to varying contents of bioactive compounds in different MC plants, perhaps as a result of minor differences in growth conditions. It is plausible to assume that inconsistencies, such as relative concentrations of sesquiterpene after 15 s as opposed to the 20 s treatment (Table 5), may be caused by agrotechnical and botanical reasons. These inconsistencies indicate that while steam sterilization had a minor effect on terpene profiles, the deviation in concentrations of these compounds in MC inflorescences due to growth conditions were much more significant^24,28^.

The role of terpenes and terpenoids for medical purposes in cannabis has not yet been resolved^27^, but has been mentioned in several recent studies (Milay et al., 2020; Russo, 2011; Russo and Marcu, 2017, Namdar et al. 2019). Regardless, terpenes and terpenoids, mostly monoterpenes, have aromatic and or medical properties that appear to play a crucial role in consumer preferences of different MC varieties^27^. Thus, observations from this study regarding the differential effects of steam sterilization on some terpenes in particular and on terpene profiles in general, is crucial.

### Effects of steam sterilization on cannabinoid content in noncommercial MC inflorescences

**Figure 1:**
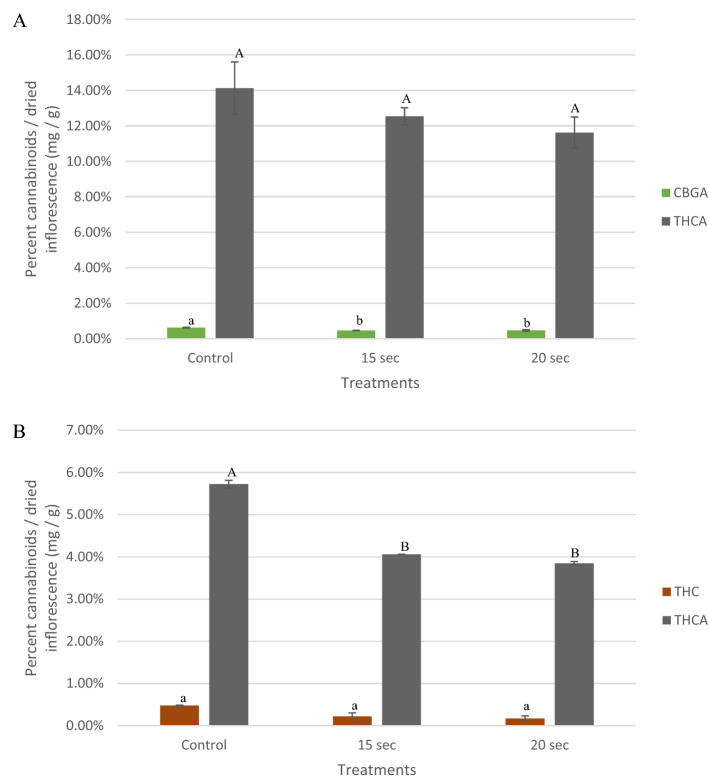
Percentage of different cannabinoids (CBGA, THC and THCA) in noncommercial inflorescences calculated as relative cannabinoid dry weight per inflorescences for the first (**A**), and second experiments (**B**). In each experiment, inflorescences were exposed to a steam treatment of 15 s at 65 °C and 20 s at 70 °C, and compared to an untreated control using two separate strains cultivated in the ARO licensed facility. Cannabinoid values at low concentrations (> 0.5%) in the untreated control are not shown. Values compared for each cannabinoid between treatments, with a different letter are significant, according to Tukey HSD (α = 0.05).

In the first experiment, a decrease in content of THCA was detected as steaming exposure levels increased (Fig. 1A), e.g. 14.12% in the untreated control compared to 12.53% and 11.62% after 15 and 20 s exposure, respectively. This decrease in concentration was insignificant according to Tukey's test at α = 0.05 (Fig. 1A). Relative amounts of the second most common cannabinoid in the samples, namely cannabigerolic acid (CBGA), decreased from 0.63% in the control to 0.47% after a 15 s treatment, but did not change after 20 s (Fig. 1A).

In the second experiment, the general pattern was similar to that of the first experiment. Percent content of THCA decreased significantly from 5.73% in the untreated control to 4.06% and 3.85%, after 15 and 20 s, respectively (Fig. 1B) while THC percentages decreased insignificantly (Fig. 1B).

Testing for changes in cannabinoid content is very challenging as the concentrations of different cannabinoids compounds may vary naturally between different inflorescences, even from the same plant^1,30^. Therefore, it is difficult to stipulate the values of permitted deviation in cannabinoid contents found in MC inflorescences. Our results were based upon definitions dictated by the United States Pharmacopeia (USP) publications stating that the allowed deviation in cannabinoids content is ± 20% of the declared percentage^1^. Additional studies may be required to determine whether the observed decreases in THCA levels following steam treatment are consistent using other noncommercial samples.

In general, in the noncommercial MC experiments, a decrease was detected in the percentage of cannabinoids during steam sterilization with an increase in exposure periods (Fig. 1). For example, in the first experiment, the 20 s treatment resulted in a decrease of 17.7% in THCA concentration compared to the untreated control (Fig. 1A) but within the 20% USP limit.

### Effects of steam sterilization on cannabinoid content in commercial MC inflorescences

**Figure 2:**
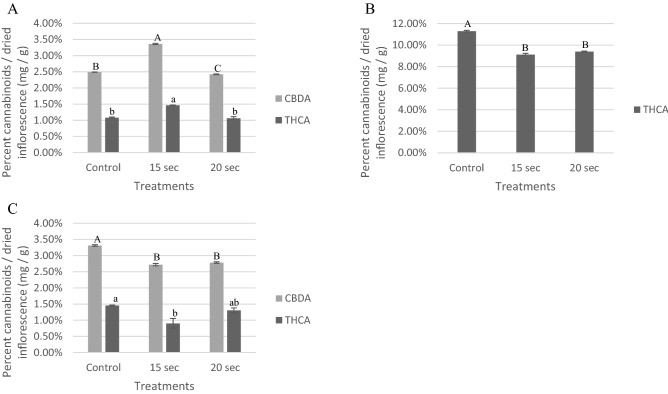
Percentage of different cannabinoids from commercial inflorescences calculated as relative cannabinoid dry weight per inflorescence for three separate experiments; A, B, and C. In each experiment, inflorescences were exposed to a steam treatment of 15 s at 65 °C and 20 s at 70 °C, and compared to an untreated control. The first (**A**) and third (**C**) experiments were conducted using inflorescences from the same commercial farm while the second experiment was conducted on inflorescences from a different farm. Cannabinoids that were present in low concentrations (> 0.5%) in the untreated control are not shown. Values compared for each cannabinoid between treatments, with a different letter are significant, according to Tukey HSD (α = 0.05).

In the commercial MC experiments, the effect of steam sterilization treatments on cannabinoid content was similar to that of the noncommercial MC inflorescences (Fig. 2). In the first experiment, concentrations of both measured cannabinoids increased after 15 s exposure but decreased after 20 s compared to that of the untreated control (Fig. 2A). For example, CBDA percentages in the control group of the first experiment measured 2.49% compared to 3.36% and 2.42% for the 15 and 20 s exposure treatments, respectively.

In contrast, in the second and third experiments, there was a decrease in percentage of measured cannabinoids in the 15 and 20 s steaming treatments, compared to the untreated sample (Figs. 2B,C). For example, in the second experiment, THCA levels of 11.29% were recorded in the untreated control compared to that of 9.12% and 9.40% in the 15 and 20 s exposure treatments, respectively (Fig. 2B).

CBN is the degradation product of THC^29^, so the fact that no increase in CBN levels was detected (supplementary Table 1 and supplementary Table 2) suggests that the observed changes in THCA levels were not caused by degradation. Still additional studies may be required to determine whether the observed decreases in THCA levels following steam treatment are consistent using other noncommercial samples.

These inconsistencies could be explained due to the sampling size in the commercial experiments that was larger than that of the noncommercial ones and resulted in more varied inflorescences from different plants of the same cultivar, thus increasing natural diversity in the cannabinoid profiles. Additional studies may be required to determine whether the observed decreases in cannabinoid levels following steam treatment are consistent using other commercial samples. It is important to note that no visible changes were observed in the inflorescences following treatments.

## Conclusions

This research demonstrated that steam sterilization reduced TYM CFU levels very effectively in MC inflorescences. An exposure period of 10 s at 62.5 ºC reduced CFUs from 10^5.58^ CFU/g inflorescence to below detection levels.

The effect of steam sterilization caused minor changes in terpene profiles. Monoterpenes extracted from MC inflorescences were reduced by the tested steam sterilization exposure times, while sesquiterpene concentrations increased. Certain terpenes, such as β-ocimene, 3-carene and endo-borneol were detected only after steaming. This should be taken into consideration and the steaming process be further tested for its effect on the therapeutic potency of the treated strains. However, additional studies are required to determine the consistency of these changes.

The effect of steam sterilization on cannabinoid contents of MC inflorescences was also examined. Although the tested steaming treatments caused a reduction in the cannabinoid profiles, for most treatments this reduction did not exceed the 20% deviation limit permitted by the USP.

## Material and methods

### Plant material

*Cannabis sativa* was cultivated in the Agriculture Research Organization (ARO) Volcani Center (authorized by the Israeli Medical Cannabis Agency, IMCA, Ministry of Health, State of Israel) for this research, as described^12^. Two different ARO cultivars, AO 235 and AO 325, comprising different profiles of cannabinoid and terpene compounds were used in the noncommercial experiments. In addition, MC inflorescences from commercial sources used in this research were supplied by two different MC farms. All experimental research on the above mentioned plants, complies with relevant institutional, national, and international guidelines and legislation.

### Steam sterilization

**Figure 3:**
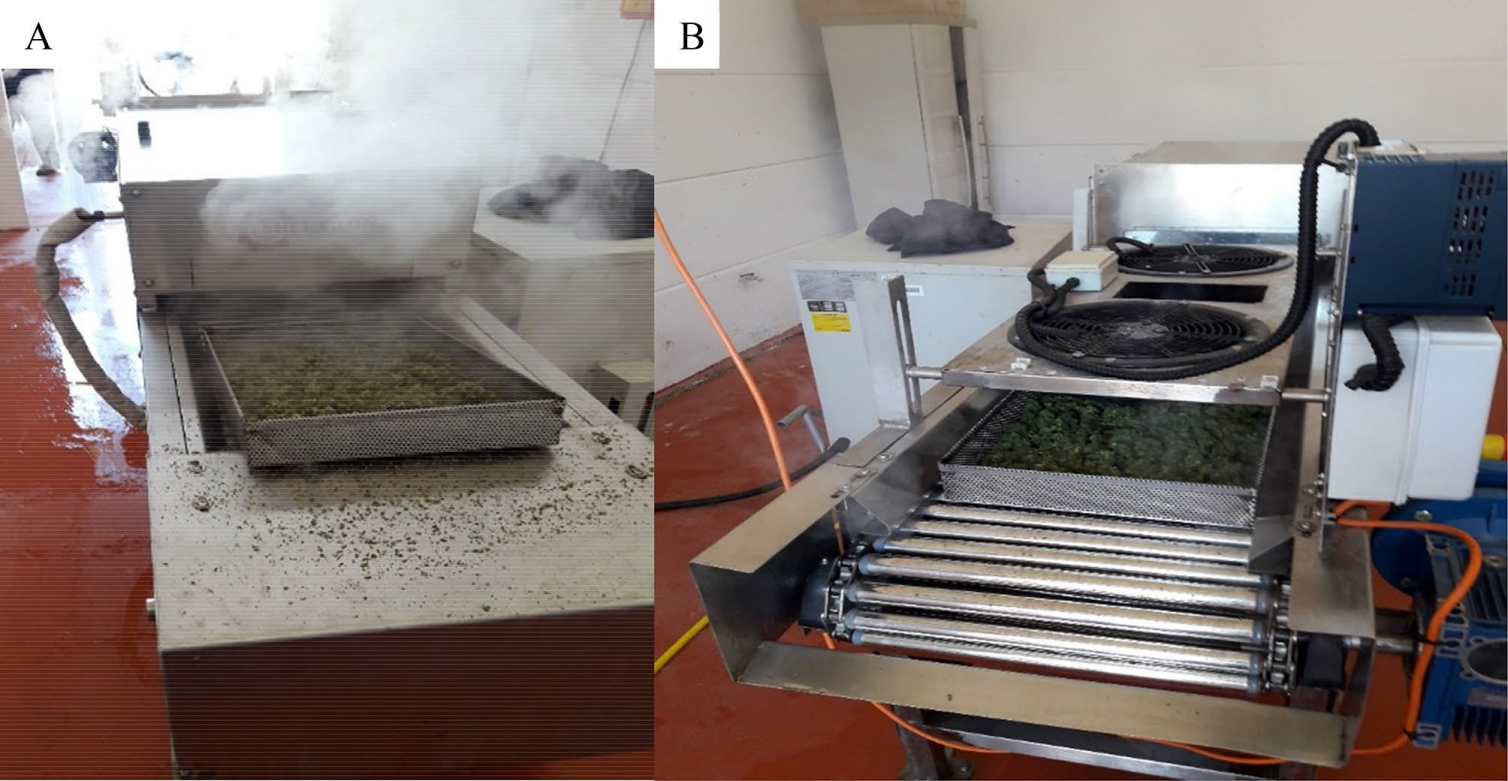
Steam sterilization prototype equipment used in this research, as shown from both ends. During the process, a stainless steel tray filled with MC inflorescences enters into the steam tunnel (**A**), exits the tunnel and is dried by the two top fans (**B**).

The steam sterilization equipment used in this experiment consisted of a rotating and advanced roller conveyor. This system is suitable for the treatment of substances and samples that can move along the rolling cylinders. Since MC inflorescences are not suitable for treatment in this manner, in our experiments we placed the inflorescences in 40 × 35 × 5 cm stainless steel trays (Fig. 3A). These trays containing the inflorescences moved on top of the cylinders into the tunnel, exposing them to 2 mm orifice emitted jets of steam. Two blowers situated adjacent to the steam tunnel were operated to dry excess moisture (Fig. 3B). The system used in these experiments is a prototype that operates on 40 kW and provides 50 kg of steam per hour. Drying the sample with air in temperature of 45 ºC during 2 min was sufficient to remove residual condensation and bring the sample to embodiment moisture levels.

For noncommercial MC experiments, 40 g inflorescences from each cultivar were sampled, 10 g per treatment. Inflorescences were exposed to steaming of 10 s at 62.5 °C, 15 s at 65 °C and 20 s at 70 °C and compared to an untreated control. Temperatures were measured within the steam chamber using a 830-T1 infrared thermometer (Testo Inc., West Chester, PA, USA). The effect of steaming was assessed for TYM CFU counts (described below), and for terpenoid and cannabinoid identification and quantification (described below). This procedure was repeated twice, with 2 different ARO cultivars, to test the effect of steaming on different cannabinoid and terpene profiles.

For commercial MC experiments 200 g inflorescences from each cultivar was sampled, 50 g per treatment. Cultivars were supplied from two different commercial MC farms in Israel. After steam treatments, 10 g inflorescences were randomly collected from each treatment and processed, as described for the noncommercial MC experiments.

### Quantification and identification of fungal colony levels

In order to determine the efficacy of steam sterilization on CFU levels in MC inflorescences, an untreated control group was compared to that of the steam treatments. Ten g of dried inflorescences from each treatment (10, 15 and 20 s of steaming, and an untreated control) were ground until homogenized mixtures were achieved. Then 1 g samples were placed into 50 ml Falcon tubes and soaked in 10 ml sterile saline solution (NaCl 0.85 g/l, Tween 20, 100 μl/l) for 10 min. This process was repeated 3 times for each treatment. One ml of liquid was removed from each Falcon tube and decimal dilutions were conducted. Thereafter, 100 µl from each sample was evenly spread on 3 PDAC [potato dextrose agar (Difco, Franklin Lakes, New Jersey, USA) supplemented with 0.25 g/l chloramphenicol] Petri plates using sterile glass beads. Plates were than kept at ambient room temperature (25° ± 2 C) for 3 to 5 days. Developing colonies were enumerated and calculated to adjust CFU concentrations to CFU/g inflorescence and the major fungal species were morphologically identified using light microscope LEICA DM500 [Leica Microsystems, Wetzlar, Germany].

### MC inflorescences extraction for terpenoid identification

Ten g of dried inflorescences from each treatment (15 and 20 s of steaming, and an untreated control) were ground until homogenized mixtures were achieved. Quantities of 1 to 5 g MC inflorescences were frozen in liquid nitrogen and ground using a porcelain mortar and pestle to achieve homogenization of the sample. The mortar and pestle were rinsed with acetone and then water, between each sample. The homogenized samples were then placed in 50 ml tubes. To each sample, 4 to 40 ml of purified *n*-Hexane (ratio of 1:8, w/v; EMSURE-grade for GC–MS; Mercury Scientific and Industrial Products Ltd. Newtown, Connecticut, United States) was added. The samples were shaken for 45 min at 220 rpm in a TU-400 orbital shaker incubator at room temperature. The supernatant containing the total extract from each sample was removed and filtered through a 0.45 mesh filter, and transferred into a sterile 20 ml scintillator glass vial. This process was repeated 3 times for each treatment. Method blanks were made (containing solvent only) routinely with each extraction batch. The samples were then dried under a gentle stream of nitrogen. No further treatment was carried out prior to separation on silica columns.

### Terpenoid identification and relative quantification using Gas Chromatography Coupled with Mass Selective Detection (GC–MS)

GC–MS analyses were carried out using an Agilent 7890B gas chromatograph (Santa Clara, CA, USA) coupled to a 5977A mass spectrometer (electron multiplier potential 2 kV, filament current 0.35 mA, electron energy 70 eV, and the spectra were recorded over the range of m/z 40 to 500), as described^24,30^. An Agilent 7683 autosampler was used for sample introduction. A 1 µl aliquot of each sample was injected into the GC–MS using a 1:10 split-ratio injection mode. Helium was used as the carrier gas at a constant flow of 1.1 ml s^−1^. An isothermal constant heat of 50 °C was maintained for 2 min, then a heating gradient of 6 °C min^−1^ to 300 °C, followed by a post-run isothermal hold at 300 °C for 8 min. A 4 min solvent delay was applied. A 30 m, 0.25 mm ID, 5% cross-linked phenylmethyl siloxane capillary column (HP-5MS) with 0.25 µm film thickness was used for separation, with an injection port temperature of 220 °C and an MS interface temperature of 280 °C. Peak assignments were analyzed with a spectral library (NIST 14.0 and 17.0) and compared with MS data obtained from the injection of a 21 terpene standard kit (Merck LTD, Teddington, UK), purchased from LGC Standards. For identification and partial quantification, 10 µg of the LGC terpenoid standards were injected to the GC–MS.

### MC inflorescence extraction for phytocannabinoid identification and quantification

Ten g of dried inflorescences from each treatment (10, 15 and 20 s of steaming, and an untreated control) were ground until homogenized mixtures were achieved. Quantities of 1 to 4 g of dry inflorescences were frozen in liquid nitrogen, ground by mortar and pestle and placed in 20 ml glass tubes. Absolute ethanol (HPLC grade, Biolab, Israel) was added to each inflorescence powder sample at a sample-to-ethanol ratio of 1:4 (w/v). The samples were mixed at 220 rpm in a TU-400 orbital shaker incubator at room temperature for 45 min, and then the extract was filtered through a 0.45 µm filter (PVDF syringe filter, Merck, Darmstadt, Germany). The extract solvent was evaporated using nitrogen until completely dry. All samples were prepared in triplicate.

The cannabinoid profile was analyzed according to a calibration curve using the following standards: cannabigerol (CBG, Restek catalog no. 34091, USA), cannabidiol (CBD, Restek catalog no. 34011), cannabidiolic acid (CBDA, Restek catalog no. 34094), cannabinol (CBN, Restek catalog no. 34010), cannabigerolic acid (CBGA, Restek catalog no. RE34112), ∆-9 tetrahydrocannabinol (Δ-9 THC, Restek catalog no. 34067), cannabichromene (CBC, Restek catalog no. 34092), tetrahydrocannabinolic acid (THCA-A, Restek catalog no. 34093) and tetrahydrocannabivarinic acid (THCVA, Sigma-Aldrich catalog no. t111). For quantification of phytocannabinoids the standards were dissolved in methanol at different concentrations from 1 to 60 ppm.

### High performance liquid chromatography (HPLC) analysis and sample separation

For analytical HPLC (1260 Infinity II, Agilent), the dry crude extract was resuspended in methanol at a concentration of 50 µg/ml and filtered through a 0.45 µm syringe filter. The filtered extract was injected to HPLC using isocratic separation with acenotrile (20%), water (80%) (HPLC grade, Biolab), including 5 mM ammonium formate, and 0.1% formic acid at a constant flow rate of 1.5 ml/min. The separation was performed on a Raptor ARC-18 2.7um 150 × 4.6 mm column (Restek, 9314A65), Injection volume of 5 µl. The compound peaks were detected at 220 and 280 nm. Quantitative determination was conducted according to the peak area at 220 nm.

### Statistical analysis

Data were processed using the JMP statistical package (https://www.jmp.com/en_us/home.html, SAS Inc, NC, USA). Comparisons between groups were made with analysis of variance (ANOVA) followed by Tukey–Kramer's honest significant difference (HSD) test as post hoc. Values are shown as mean ± standard error (s.e.m.). *P* values ≤ 0.05 were considered significant.

## Supplementary Information

Supplementary Information 1.

Supplementary Information 2.
