# Supplementary material for: Effects of steam sterilization on reduction of fungal colony forming units, cannabinoids and terpene levels in medical cannabis inflorescences
Source: Sci Rep. 2021 Jul 7;11:13973. doi: 10.1038/s41598-021-93264-y (PMC8263730; doi:10.1038/s41598-021-93264-y)
Supplement: Supplementary file 1 — Supplementary Information 1. [file 41598_2021_93264_MOESM1_ESM.docx]

Supplementary Table 1. Results of all 14 cannabinoids tested in uncommercial samples.

|  |  | S0 | | S15 | | S20 | |
| --- | --- | --- | --- | --- | --- | --- | --- |
|  | Cannabinoid | Cannabinoid content (%) | Standard error | Cannabinoid content (%) | Standard error | Cannabinoid content (%) | standard error |
| First uncommercial | CBDVA | -^a^ |  | - |  | - |  |
|  | CBC | - |  | - |  | - |  |
|  | CBCA | - |  | - |  | - |  |
|  | CBD | - |  | - |  | - |  |
|  | CBDA | - |  | - |  | - |  |
|  | CBDV | - |  | - |  | - |  |
|  | CBG | - |  | - |  | - |  |
|  | CBGA | 0.6253 | 0.015 | 0.466 | 0.006 | 0.474 | 0.0351 |
|  | CBL | - |  | - |  | - |  |
|  | CBN | - |  | - |  | - |  |
|  | THC | 0.3687 | 0.02 | 0.3487 | 0.0042 | 0.3397 | 0.0506 |
|  | THCA | 14.122 | 1.4783 | 12.5313 | 0.4943 | 11.6183 | 0.8796 |
|  | THCV | - | - | - | - | - | - |
|  | THCVA | - | - | - | - | - | - |
| Second uncommercial | CBDVA | - |  | - |  | - |  |
|  | CBC | - |  | - |  | - |  |
|  | CBCA | - |  | - |  | - |  |
|  | CBD | - |  | - |  | - |  |
|  | CBDA | - |  | 0.044 | 0.044 | - |  |
|  | CBDV | - |  | - |  | - |  |
|  | CBG | - |  | 0.0317 | 0.0317 | - |  |
|  | CBGA | 0.2597 | 0.0305 | 0.1967 | 0.0018 | 0.268 | 0.056 |
|  | CBL | - |  | - |  |  |  |
|  | CBN | - |  | - |  |  |  |
|  | THC | 0.4783 | 0.0093 | 0.2237 | 0.0814 | 0.173 | 0.0605 |
|  | THCA | 5.7263 | 0.0906 | 4.0617 | 0.0039 | 3.8487 | 0.0393 |
|  | THCV | - |  | - |  | - |  |
|  | THCVA | - |  | - |  | - |  |
| ^a^ Represent cannabinoid that were not detected at all during the measurements. | | | | | | | |
