# Supplementary material for: Effects of steam sterilization on reduction of fungal colony forming units, cannabinoids and terpene levels in medical cannabis inflorescences
Source: Sci Rep. 2021 Jul 7;11:13973. doi: 10.1038/s41598-021-93264-y (PMC8263730; doi:10.1038/s41598-021-93264-y)
Supplement: Supplementary file 2 — Supplementary Information 2. [file 41598_2021_93264_MOESM2_ESM.docx]

Supplementary Table 2. Results of all 14 cannabinoids tested in commercial samples.

|  |  | S0 | | S15 | | S20 | |
| --- | --- | --- | --- | --- | --- | --- | --- |
| First commercial | Cannabinoid | Cannabinoid content (%) | Standard error | Cannabinoid content (%) | Standard error | Cannabinoid content (%) | Standard error |
|  | CBDVA | -^a^ |  | - |  | - |  |
|  | CBC | - |  | - |  | - |  |
|  | CBCA | - |  | - |  | - |  |
|  | CBD | - |  | - |  | - |  |
|  | CBDA | 3.311 | 0.026 | 2.719 | 0.037 | 2.784 | 0.024 |
|  | CBDV | - |  | - |  | - |  |
|  | CBG | - |  | - |  | - |  |
|  | CBGA | - |  | - |  | - |  |
|  | CBL | - |  | - |  | - |  |
|  | CBN | - |  | - |  | - |  |
|  | THC | - |  | - |  | - |  |
|  | THCA | 1.455 | 0.012 | 0.902 | 0.152 | 1.305 | 0.075 |
|  | THCV | - |  | - |  | - |  |
|  | THCVA | - |  | - |  | - |  |
| Second commercial | CBDVA | - |  | - |  | - |  |
|  | CBC | - |  | - |  | - |  |
|  | CBCA | - |  | - |  | - |  |
|  | CBD | - |  | - |  | - |  |
|  | CBDA | - |  | - |  | - |  |
|  | CBDV | - |  | - |  | - |  |
|  | CBG | - |  | - |  | - |  |
|  | CBGA | 0.28467 | 0.02772 | 0.255 | 0.00555 | 0.04776 | 0.04776 |
|  | CBL | - |  | - |  | - |  |
|  | CBN | - |  | - |  | - |  |
|  | THC | 0.42067 | 0.07604 | 0.369 | 0.14889 | 0.325 | 0.11628 |
|  | THCA | 11.29 | 0.08249 | 9.12 | 0.11477 | 9.4 | 0.04323 |
|  | THCV | - |  | - |  | - |  |
|  | THCVA | - |  | - |  | - |  |
| Third commercial | CBDVA | - |  | - |  | - |  |
|  | CBC | - |  | - |  | - |  |
|  | CBCA | - |  | - |  | - |  |
|  | CBD | 0.041 | 0.0215 | 0.1107 | 0.0058 | 0.0757 | 0.0067 |
|  | CBDA | 2.49 | 0.004 | 3.3623 | 0.0185 | 2.4213 | 0.0123 |
|  | CBDV | - |  | - |  | - |  |
|  | CBG | - |  | - |  | - |  |
|  | CBGA | 0.0853 | 0.0066 | 0.096 | 0.0175 | 0.0633 | 0.013 |
|  | CBL | - |  | - |  | - |  |
|  | CBN | - |  | - |  | - |  |
|  | THC | 0.0543 | 0.0274 | 0.0787 | 0.0239 | 0.0317 | 0.016 |
|  | THCA | 1.0807 | 0.02 | 1.4627 | 0.0082 | 1.063 | 0.0477 |
|  | THCV | - |  | - |  | - |  |
|  | THCVA | - |  | - |  | - |  |
| ^a^ Represent cannabinoid that were not detected at all during the measurements. | | | | | | | |
